# Cystic Subcutaneous Phaeohyphomycosis Caused by Cladophialophora Species in an Elderly Leprosy Patient

**DOI:** 10.7759/cureus.71287

**Published:** 2024-10-12

**Authors:** P B Praveen Kumar, M Mala, Eunice Swarna Jacob

**Affiliations:** 1 Department of Microbiology, Thanjavur Medical College, Thanjavur, IND

**Keywords:** cladophialophora, cystic subcutaneous phaeohyphomycosis, fluconazole, leprosy, one celled blastoconidia

## Abstract

Phaeoid (dematiaceous) fungi are a diverse group of species characterized by their production of the pigment dihydroxynaphthalene melanin. Although phaeoid fungi do not usually cause human infections, they are commonly found in nature as contaminants. These fungi are present in decomposing vegetation, rotting timber, and soil. However, an increase in infections is probably due to the increase in the population of individuals with compromised immune systems. An elderly female patient from the southernmost part of India, who was previously treated for leprosy, presented with multiple boggy swellings in her right hand. An appropriate sample was collected under aseptic precautions and subjected to microbiological analysis, which led to the isolation of *Cladophialophora* species. The patient was treated with antifungal drugs, but her condition worsened, resulting in a poor outcome.

## Introduction

Leprosy, sometimes referred to as Hansen's disease, is an infectious disease that is treatable but is still widespread in more than 140 countries worldwide [1]. In leprosy patients, the most commonly reported non-viral coinfections are tuberculosis, leishmaniasis, chromoblastomycosis-like infections, and helminths. Even though they are very common, coinfections such as leprosy and chromoblastomycosis-related infections are rare [2]. Cystic subcutaneous phaeohyphomycosis is a localized cutaneous infection following deep inoculation of fungus into subcutaneous tissues. The lesions are found on the feet, legs, hands, arms, back, or other body sites. The nodules develop as indolent, usually small subcutaneous masses [3].

In this report, we present the case of cystic subcutaneous phaeohyphomycosis caused by *Cladophialophora *species in a diabetic woman who recovered from Hansen’s disease 50 years ago. The pathogen was identified as *Cladophialophora *species by demonstrating characteristic colony morphology and microscopic features. The patient was treated with fluconazole, which resulted in an unfavorable outcome. Increased awareness is necessary for the appropriate treatment of such conditions. *Cladophialophora *species are highly neurotropic and often prove fatal, hence are frequently isolated from brain abscesses. This species has been involved in human skin lesions along with cases of chromoblastomycosis-like infections and has even recently been reported as a lung infection [3].

This article was previously presented as a poster at the Sai MicroCon 2023 International E-Conference on July 20, 2023.

## Case presentation

**Figure 1:**
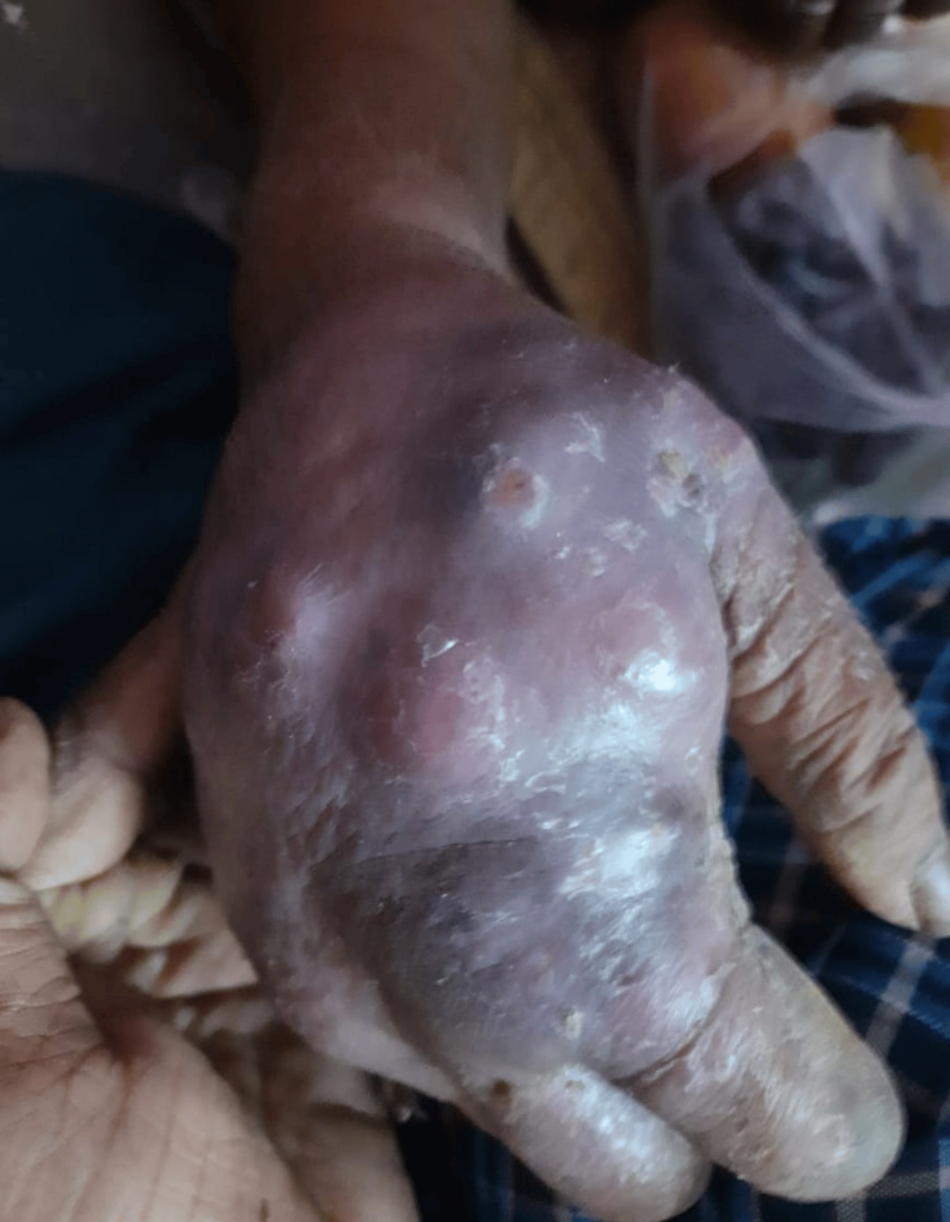
Multiple boggy swellings in the patient's right hand (upper view)

**Figure 2:**
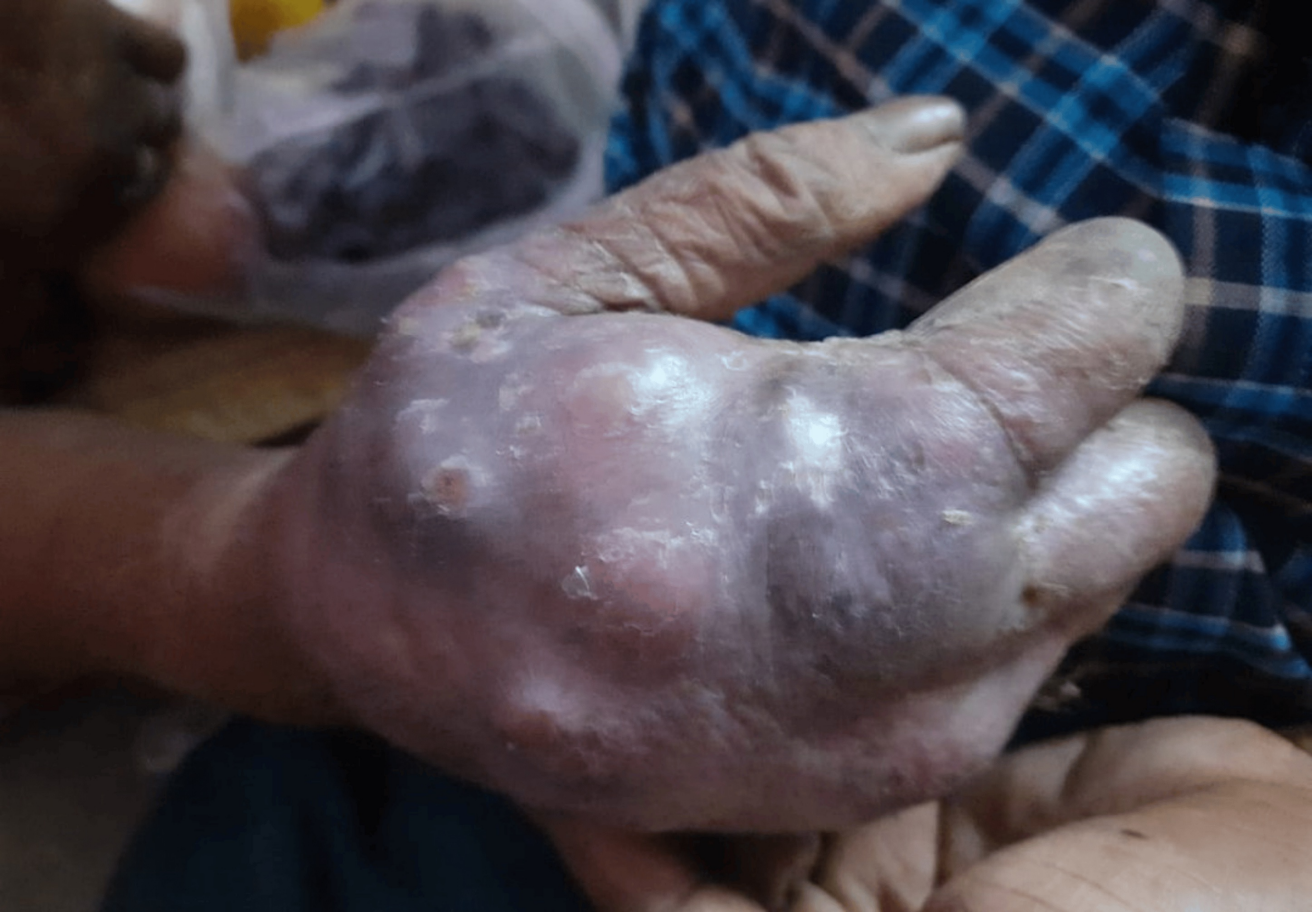
Multiple boggy swellings in the patient's right hand (side view)

An 85-year-old woman, with an agricultural background, known to be diabetic on irregular treatment, who had recovered from Hansen’s disease approximately 50 years ago, presented with complaints of multiple boggy swellings (Figures 1, 2) in her right hand. The onset was insidious and progressive in nature over the past two months. There was no history of numbness, sensory deficit in any of the four limbs, trauma, or fever.

**Figure 3:**
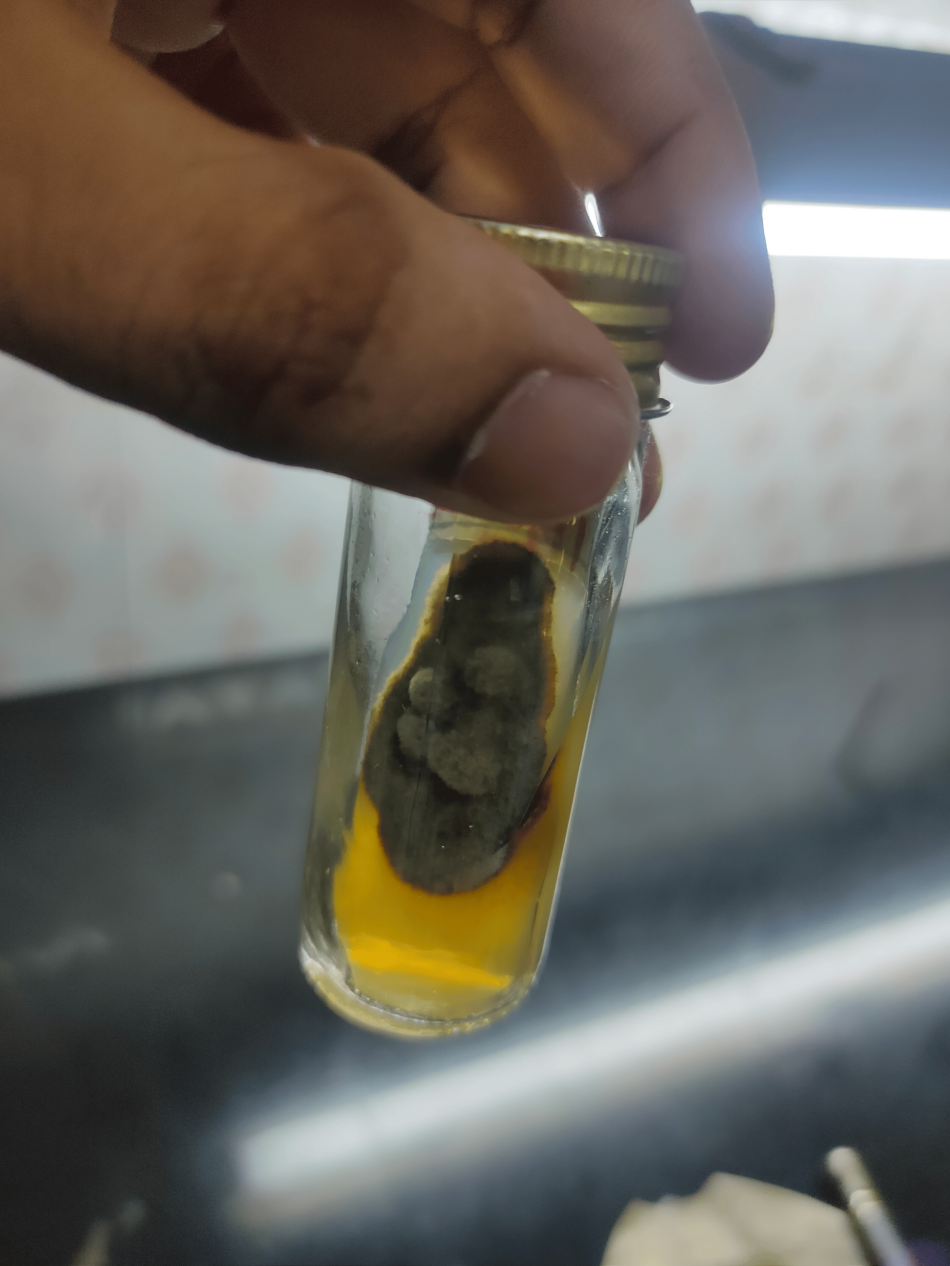
Sabouraud dextrose agar with chloramphenicol (obverse)

**Figure 4:**
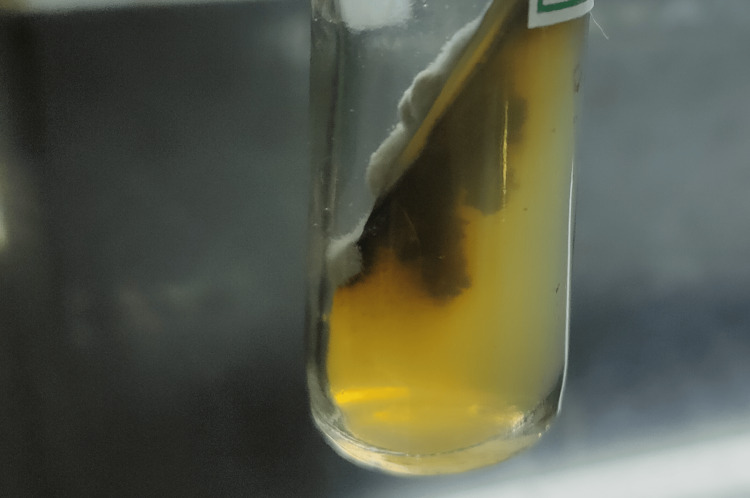
Sabouraud dextrose agar with chloramphenicol (reverse)

A pus aspirate sample was collected from the boggy swelling and sent for fungal examination. Potassium hydroxide (KOH) mount revealed numerous irregular septate-branched hyphae [3]. Sabouraud dextrose agar (SDA) with chloramphenicol showed spreading growth, olive-gray to brown in color (Figure 3), and the reverse was black as shown in Figure 4 [4].

**Figure 5:**
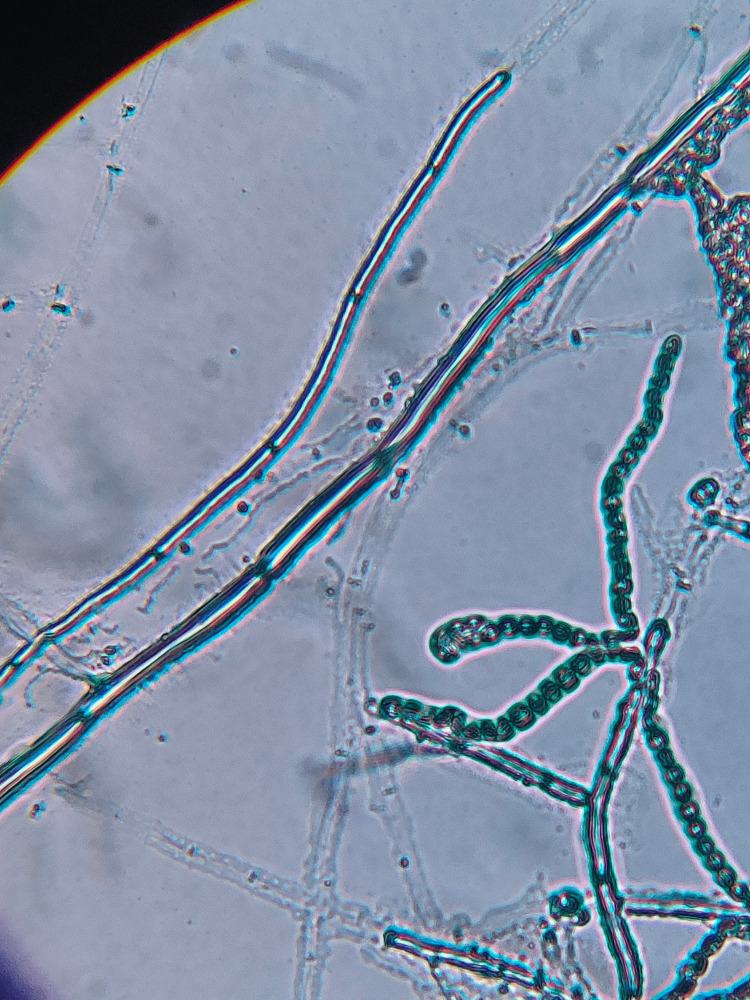
Lactophenol cotton blue (LPCB) mount

Lactophenol cotton blue (LPCB) mount revealed one-celled blastoconidia with no evident darkly pigmented hila, which are borne in long, sparsely branched conidial chains (Figure 5) [3].

**Figure 6:**
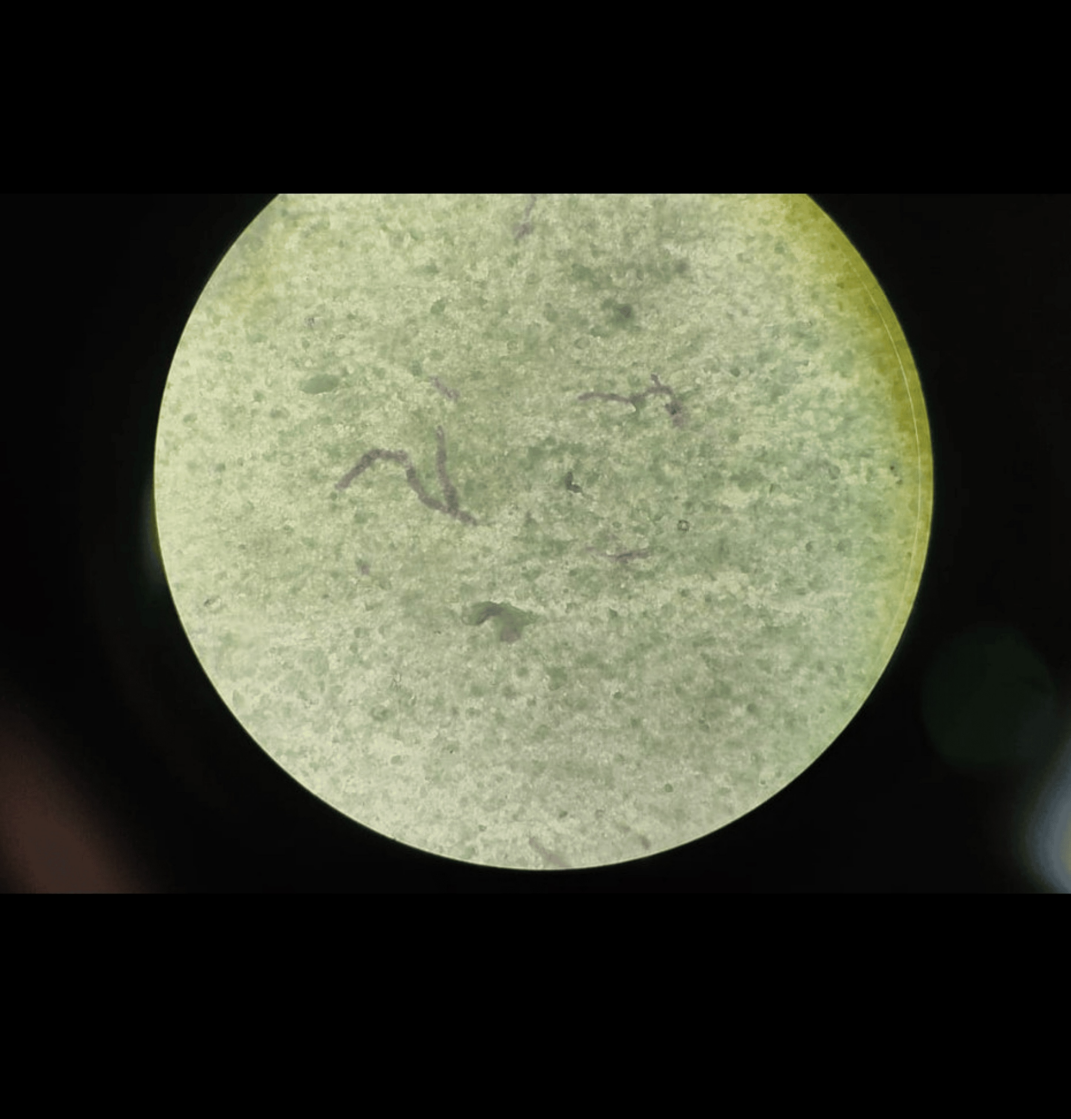
Periodic acid-Schiff (PAS) stain Light green is used as a counterstain.

**Figure 7:**
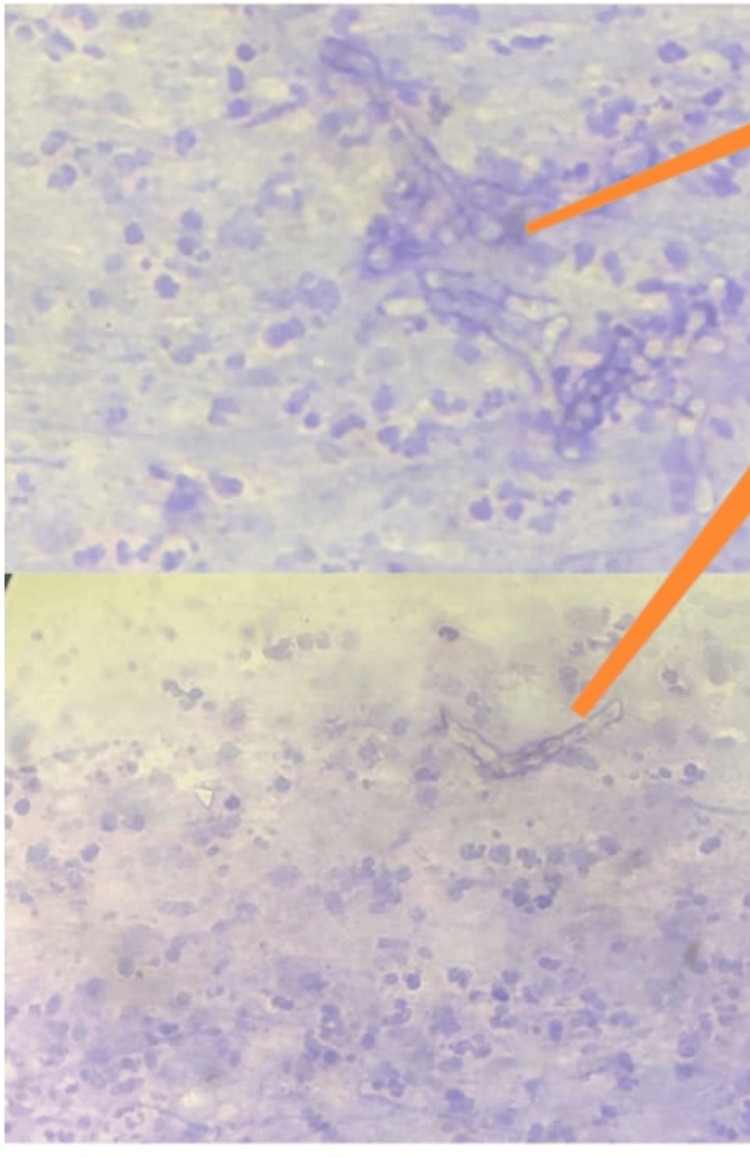
Periodic acid-Schiff (PAS) stain Hematoxylin is used as a counterstain.

Periodic acid-Schiff (PAS) stain revealed sheets of neutrophils admixed with eosinophils and lymphocytes in a necrotic background. Thin septate-branched fungal hyphae were observed (Figures 6, 7).

Other relevant biochemical investigations were conducted, including complete blood count, renal function tests, liver function tests, lipid profile, thyroid profile, HbA1c, chest X-ray, and computed tomography (CT) scan of the brain. All reports were within normal limits. The patient was also non-reactive for human immunodeficiency virus (HIV), hepatitis B surface antigen (HBsAg), and antibodies to hepatitis C virus (Anti-HCV) tests.

Based on the clinical picture and microbiological analysis, we diagnosed this case as cystic subcutaneous phaeohyphomycosis, and the isolate was presumptively identified as *Cladophialophora *species. Due to the non-availability of effective medical treatment, early diagnosis and excision of the lesion are indicated to avoid further complications [3]. However, we proceeded with antifungal therapy, considering factors such as the patient's age, immune status, and comorbidities, but this resulted in a poor prognosis and a negative outcome.

## Discussion

Suspicion of a fungal infection arises when a patient presents with multiple boggy swellings and comorbidities like diabetes mellitus and does not respond to the usual empirical therapy. Besides the well-known phaeoid fungi, species such as *Phaeoacremonium minimum*, *Exophiala jeanselmei*, *Pleurostomophora richardsiae*, and *Exophiala salmonis *are reported as human pathogens causing cystic subcutaneous phaeohyphomycosis [5-8]. The successful outcome of *Cladophialophora *infection is related to underlying disease control and appropriate antifungal therapy like liposomal amphotericin B and azole derivatives such as voriconazole [3].

## Conclusions

*Cladophialophora *species has a predilection for the central nervous system and consequently causes cerebral phaeohyphomycosis. However, some reports have implicated this fungus as an agent of subcutaneous phaeohyphomycosis and infections in other systems, such as the lungs. Therefore, increasing knowledge about the various species that cause subcutaneous mycosis is essential. Due to the emergence of antifungal resistance, effective preventive measures and awareness of fungal species are urgently needed to curtail these infections.
